# Tracking synthesis and turnover of triacylglycerol in leaves

**DOI:** 10.1093/jxb/eru500

**Published:** 2015-01-21

**Authors:** Henrik Tjellström, Merissa Strawsine, John B. Ohlrogge

**Affiliations:** Department of Plant Biology and Department of Energy, Great Lakes Bioenergy Research Center Michigan State University, East Lansing, MI 48824, USA

**Keywords:** Acyl-CoA, DGAT, diacylglycerol acyltransferase, leaf TAG, lipids, triacylglycerol.

## Abstract

Triacylglycerol (TAG), typically represents <1% of leaf glycerolipids but can accumulate under stress and other conditions or if leaves are supplied with fatty acids, or in plants transformed with regulators or enzymes of lipid metabolism. To better understand the metabolism of TAG in leaves, pulse–chase radiolabelling experiments were designed to probe its synthesis and turnover. When *Arabidopsis* leaves were incubated with [^14^C]lauric acid (12:0), a major initial product was [^14^C]TAG. Thus, despite low steady-state levels, leaves possess substantial TAG biosynthetic capacity. The contributions of diacylglycerol acyltransferase1 and phospholipid:diacylglycerol acyltransferase1 to leaf TAG synthesis were examined by labelling of *dgat1* and *pdat1* mutants. The *dgat1* mutant displayed a major (76%) reduction in [^14^C]TAG accumulation whereas *pdat1* TAG labelling was only slightly reduced. Thus, DGAT1 has a principal role in TAG biosynthesis in young leaves. During a 4h chase period, radioactivity in TAG declined 70%, whereas the turnover of [^14^C]acyl chains of phosphatidylcholine (PC) and other polar lipids was much lower. Sixty percent of [^14^C]12:0 was directly incorporated into glycerolipids without modification, whereas 40% was elongated and desaturated to 16:0 and 18:1 by plastids. The unmodified [^14^C]12:0 and the plastid products of [^14^C]12:0 metabolism entered different pathways. Although plastid-modified ^14^C-labelled products accumulated in monogalactosyldiacylglycerol, PC, phosphatidylethanolamine, and diacylglcerol (DAG), there was almost no accumulation of [^14^C]16:0 and [^14^C]18:1 in TAG. Because DAG and acyl-CoA are direct precursors of TAG, the differential labelling of polar glycerolipids and TAG by [^14^C]12:0 and its plastid-modified products provides evidence for multiple subcellular pools of both acyl-CoA and DAG.

## Highlight

Analysis of flux of exogenously supplied [^14^C]fatty acid in wild-type and mutant *Arabidopsis* leaves indicates that DGAT1 is the predominant enzyme involved in triacylglycerol synthesis in young leaves.

## Introduction

Almost all plant oils are currently derived from seed or seed-associated tissues such as the oil palm mesocarp. Consumption of edible plant oils is expected to double over the next 30 years in response to increasing world population and rising incomes (Chapman and Ohlrogge, 2012). In addition to utilization as food, the versatility of plant oils for many non-food uses contributes to growing demands. Therefore, development of additional strategies to produce plant oils will meet a number of societal needs. One strategy to increase the supply of plant oil is to engineer production in vegetative tissues (Durrett *et al.*, 2008; Sanjaya *et al.*, 2011; Chapman *et al.*, 2013; Vanhercke *et al.*, 2013, 2014). Oils are easily extracted from plant tissues, and residual biomass can be used for biofuels via fermentation, pyrolysis, or by burning to produce bioelectricity (Ohlrogge *et al.*, 2009). Higher oil content in vegetative tissues can also increase the nutritional value of fodder crops (Winichayakul *et al.*, 2013). In general, very minor amounts of oil are found in leaves, stems, and roots, and these amounts decrease in leaves during plant senescence (Yang and Ohlrogge, 2009). Environmental effects, such as ozone exposure and nitrogen deprivation, appear to stimulate plant oil accumulation in leaves (Sakaki *et al.*, 1990; Lippold *et al.*, 2012). In addition, several recent studies have demonstrated that a variety of molecular/genetic strategies can lead to triacylglycerol (TAG) accumulation in leaves, at up to 15% of dry weight (DW) (Sanjaya *et al.*, 2011; Fan *et al.*, 2013; Vanhercke *et al.*, 2013, 2014; Winichayakul *et al.*, 2013).

In plants, TAG is synthesized by acylation of diacylglycerol (DAG) by diacylglycerol acyltransferase (DGAT) or phospholipid:diacylglycerol acyltransferase (PDAT) using acyl-CoA or phospholipid as acyl donor, respectively (Katavic *et al.*, 1995; Routaboul *et al.*, 1999; Dahlqvist *et al.*, 2000; Zhang *et al.*, 2009). *Arabidopsis* mutants of DGAT1 and double mutants of DGAT1/PDAT1 demonstrate that these two enzymes are responsible for the majority of seed TAG synthesis (Zhang *et al.*, 2009), but their contribution to TAG synthesis in leaves is less clear. The level of TAG DW^–1^ is ~500-fold higher in *Arabidopsis* seeds compared with leaves (350 μg mg versus 0.6 μg mg DW^–1^; Y. Li *et al.*, 2006; Yang and Ohlrogge, 2009; Sanjaya *et al.*, 2013). In contrast, DGAT1 transcripts are only 5-fold more abundant in seed compared with leaves (Winter *et al.*, 2007). Thus, transcript levels suggest that leaves may have inherent capacity for synthesizing TAG, and indeed DGAT activity has been measured in leaves (Martin and Wilson, 1983). An *Arabidopsis dgat1* mutant displayed a 10-fold reduction of leaf TAG compared with the wild type (WT) in 4-week-old plants (Slocombe *et al.*, 2009), although a reduction of TAG in the *dgat1* mutant was not observed by Fan *et al.* (2013). Differences have also been observed from overexpression of DGAT1 in *Arabidopsis* and *Nicotiana* leaves (Fan *et al.*, 2013; Vanhercke *et al.*, 2013).

The *Arabidopsis pdat1* mutant has no seed oil phenotype (Mhaske *et al.*, 2005); however, *in vitro* assays suggest that PDAT contributes 10–60% of TAG synthesis in sunflower and safflower microsomes (Banaś *et al*., 2013). Notably, AtPDAT1 is more highly expressed in *Arabidopsis* leaves than seeds (Winter *et al.*, 2007), but the role of PDAT in leaves is largely unknown, although it has been suggested that it may have a role in removing oxygenated fatty acid (FA) from membrane lipids (Banaś *et al*., 2014). Studies on the overexpression of 35S-PDAT1 in *Arabidopsis* have also resulted in different results (Ståhl *et al.*, 2004; Fan *et al.*, 2013; Banaś *et al*., 2014). Ståhl *et al*. (2004) reported no changes in lipid phenotype in *Arabidopsis* seedlings between 35S-PDAT1 and control plants. More recently, Fan *et al.* (2013) reported up to 8.7% TAG DW^–1^ in *tgd1* mutants transformed with 35S-AtPDAT1/35S-OLEOSIN1. However, Banaś and co-workers did not observe TAG accumulation in WT *Arabidopsis* transformed with 35S-PDAT, although these plants had increased growth and biomass, resulting in increased FA content on a plant basis (Banaś *et al*., 2014). Thus, the involvement of PDAT1 in leaf lipid metabolism remains uncertain, particularly in non-transformed plants.

In addition to DGAT1 and PDAT1, it is notable and intriguing that *Arabidopsis* possesses at least six other genes that encode proteins reported to transfer acyl groups to DAG: two diacylglycerol acyltransferases (DGAT2 and DGAT3) (Saha *et al.*, 2006; Hernández *et al.*, 2012; Zhou *et al.*, 2013), Defective in Cuticular Ridges (DCR) (Panikashvili *et al.*, 2009; Rani *et al.*, 2010), phytyl ester synthase (PES) 1 and 2 (Lippold *et al.*, 2012), and the bifunctional wax synthase/diacylglycerol acyltransferase (WSD1) (Bird *et al.*, 2007; Li *et al.*, 2008).

Although data of Zhang *et al.* (2009) indicate that DGAT1 and PDAT1 account for the majority of TAG synthesis in seeds, the possible contribution of these other acyltransferase enzymes to TAG biosynthesis in leaves is largely unknown. As an initial approach to assess possible roles of different *Arabidopsis* acyltransferases in leaf TAG synthesis, this study has examined the ability of two acyltransferases mutants to synthesize TAG.

Radiolabelling studies with a range of precursors including acetate, CO_2_, and free fatty acids (FFAs) have been one of the most important tools to uncover plant lipid metabolic pathways (Roughan and Slack, 1982). Leaves are able to incorporate and metabolize FFA that is applied directly to the leaf surface, fed through the petiole, or provided in an aqueous buffer (Stobart *et al.*, 1980; Thompson and Roughan, 1986; Roughan *et al.*, 1987; Koo *et al.*, 2005). Surprisingly, although TAG is a very minor component of leaves, earlier reports indicated that TAG is one of the major glycerolipids synthesized from exogenously supplied FFA (Thompson and Roughan, 1986; Roughan *et al.*, 1987; Koo *et al.*, 2005). To better understand metabolic pathways involved in leaf TAG synthesis and turnover, pulse–chase radiolabelling of leaves with [^14^C]FA was used here. This methodology allowed a kinetic analysis of the synthesis and turnover of leaf TAG and other lipids, as well as information on the role of candidate acyltransferases enzymes in leaf TAG synthesis. In addition, different patterns of labelling of glycerolipid products provided evidence for the occurrence of multiple pools of extraplastidial acyl-CoA and DAG.

## Materials and methods

### Plant cultivation

*Arabidopsis* lines Col-0 (referred to as the WT), *dgat1* (Routaboul *et al.*, 1999)*, pdat1* (Mhaske *et al.*, 2005), and *pxa1* (Zolman *et al.*, 2001) were grown in 120–150 μE m^−2^ s^−1^ with a 18/6h and 23/19 °C day/night regime. Col-0, *dagt1*, and *pdat1* were grown on soil, and *pxa1* was germinated on Murashige and Skoog (MS) plates supplemented with 1% sucrose and transplanted to soil after 7 d.

### Radiolabelling

Three-week-old *Arabidopsis* leaves were transferred to a Petri dish containing 30ml of 25mM MES-KOH pH5.7 supplemented with 0.01% Triton X-100 as wetting agent. Radiolabelling was initiated by the addition of 5 μCi of [1-^14^C]lauric acid (specific activity 50 mCi mmol^–1^). Incubations were performed with gentle agitation (60rpm) under light (70 μE m^−2^ s^−1^). After 60min, the remaining leaves were rinsed in 25mM MES-KOH pH 5.7 and transferred to 25mM MES-KOH pH 5.7 medium (without wetting agent and radiolabel) and incubation continued an additional 4h to provide the ‘chase’ period.

### Lipid extraction

At the time points indicated in the figures, two leaves were transferred to 3ml of boiling isopropanol and heated to 80 °C for 10min. Isopropanol was evaporated under nitrogen gas, and 2ml of methanol:chloroform:water (10:5:4) was added. Samples were incubated for 30min in darkness (to prevent chlorophyll loss) and lipids were extracted according to Bligh and Dyer (1959). Samples were resuspended in 3ml of chloroform:methanol (2:1).

### Thin-layer chromatography (TLC)

Neutral lipids were separated on normal phase TLC plates (Uniplate silica gel HL 250 μm Analtech) using hexane:diethylether:acetic acid (70:30:1) as the solvent system. Polar lipids were separated on ammonia-impregnated normal phase TLC plates using acetone:toluene:water (91:30:8, v/v/v) as the mobile phase. Lipids were identified by co-chromatography with commercial lipid standards.

### [^14^C]FAME analysis

To determine the [^14^C]FA composition of glycerolipids and FFAs, fatty acid methyl esters (FAMEs) were prepared according to Y. Li *et al.* (2006). To each lipid band, 200 μg of tri12:0TAG were added as a carrier to minimize [^14^C]FAME losses during the transmethylation procedure. Lipid bands were scraped off TLC plates into a screw-cap tube and 300 μl of toluene and 2ml of 5% H_2_SO_4_ in methanol were added. Samples were heated for 90min at 80 °C. The resulting FAMEs were extracted with hexane and evaporated to dryness under nitrogen gas. FAMEs were separated by reverse-phase TLC (Partsil KC18 silica gel 250 μm, Whatman) using acetonitrile:methanol:acetic acid (65:30:0.5) as mobile phase or by argentation TLC as previously described (Cahoon and Ohlrogge, 1994). For argentation TLC, Partsil K6 TLC plates, 250 μm, were impregnated in 15% (w/v) AgNO_3_ in acetonitrile for 10min with gentle agitation and were left to dry in darkness overnight. Argentation TLC plates were triple developed in toluene at –20 °C. FAMEs were identified by co-chromatography of FAMEs generated from commercial [^14^C]FFAs.

### [^14^C]Molecular species analysis

Molecular species separation of DAG and phosphatidylcholine (PC) by argentation TLC is most reliable after the polarity of these lipids is reduced by conversion to TAG (Christie, 2003). [^14^C]PC, DAG, and TAG were separated by TLC as above. A 200 μg aliquot of non-labelled PC, DAG, or TAG was added as carrier to the respective lipids to minimize losses of ^14^C-labelled lipids. After TLC, bands were scraped into a test tube and incubated for 30min with 2ml of chloroform:methanol:water (5:5:1) to extract the lipids from the silica gel. Phase partition was then induced by the addition of 1ml of chloroform and 800 μl of 0.88% KCl. The lower phase was dried under nitrogen gas. For PC analysis, in order to achieve molecular species analysis by argentation TLC, the isolated lipid was reacted with 5U of phospholipase C from *Bacillus cereus* to produce DAG. Incubation periods lasted 3h with vigorous agitation in 1ml of 0.1M borate buffer (pH 7.5) and 1ml of diethyl ether. The resulting DAG product was extracted three times with 4ml of diethyl ether. To convert to TAG, the isolated DAG and the DAG moiety from PC were acetylated in 100 μl of pyridine and 150 μl of acetic anhydride for 60min at 60 °C. The resulting acetylated [^14^C]DAG and [^14^C]TAG molecular species were separated by argentation TLC as previously described (Bates *et al.*, 2009).

### Quantification of radiolabelled lipids

Absolute amounts of radioactivity (dpm) were measured by scintillation counting. The relative distribution of radioactivity between different lipids was measured by exposing TLC plates to phosphoimager screens (Imaging Screen-K, Kodak), imaged using Personal Molecular imager (Bio Rad), and quantified using the accompanying software. Radiolabel incorporation is presented relative to chlorophyll determined according to Arnon (1949).

## Results and Discussion

### Exogenous free fatty acids are rapidly incorporated into triacylglycerol and major glycerolipids in *Arabidopsis* leaves

To better understand the possible mechanisms of leaf TAG synthesis, kinetic pulse–chase labelling experiments were designed to monitor the incorporation of FAs into leaf TAG and other cellular lipids. [^14^C]12:0, [^14^C]16:0, and [^14^C]18:1 FFAs were initially tested and all were incorporated into TAG (Supplementary Fig. S1 available at *JXB* online). [^14^C]12:0 was selected as the substrate for pulse–chase experiments because of its more rapid overall incorporation into glycerolipids and its greater solubility, which facilitates removal of the label during the chase period. [^14^C]12:0 can be activated in the cytosol by long chain acyl-CoA synthetase and incorporated into glycerolipids without modification. In addition, [^14^C]12:0 can enter chloroplasts, where it is activated to acyl-ACP and elongated by *de novo* FA synthesis prior to incorporation into glycerolipids (Koo *et al.*, 2005). Therefore, an additional advantage of using [^14^C]12:0 for labelling is that it provides the ability to trace simultaneously cytosolic glycerolipid synthesis from unmodified [^14^C]12:0 as well as from [^14^C]FAs exported from plastids after *de novo* elongation of [^14^C]12:0. Experiments were conducted with a 60min pulse, after which [^14^C]12:0 was removed; analysis of the radiolabelled lipids continued for an additional 4h chase.

**Fig. 1.**
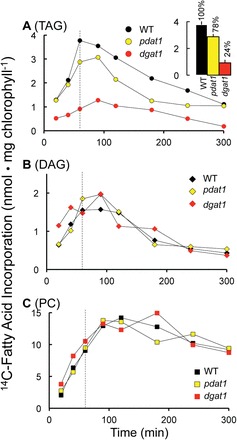
Synthesis and turnover of triacylglycerol in leaves of the wild type (WT) and *dgat1* and *pdat1* mutants. Incorporation of [^14^C]fatty acids into triacylglycerols (A), diacylglycerol (B), and phosphatidylcholine (C) in *Arabidopsis* WT and *pdat1* and *dgat1* mutants. The dotted line represents the end of the pulse period and the beginning of the chase period. The inset graph presents the percentage of WT incorporation after 60min of labelling. The data represent the average of three biological replicates. Additional data including all identified glycerolipids and free fatty acids with error bars are presented in Supplementar Fig. S2 at *JXB* online. (Figure 1 is available in colour at *JXB* online.)

Supplying [^14^C]12:0 to *Arabidopsis* leaves resulted in incorporation of radioactivity into all major glycerolipid classes (Fig. 1; Supplementary Figs S2, S3 at *JXB* online). At the earliest sampling time (20min), TAG was one of the two most highly labelled lipid classes, with a level similar to [^14^C[PC. Therefore, although TAG represents <1% of the glycerolipids in *Arabidopsis* leaves, the initial incorporation of ^14^C into TAG confirms that young leaf tissue has a high capacity for TAG synthesis without expression of additional genes.

### Contribution of DGAT1 and PDAT1 to TAG synthesis in leaves

To investigate the roles of DGAT1 and PDAT1 in the rapid accumulation of TAG, labelling of the WT was compared with that of *dgat1* and *pdat1* mutants. All three *Arabidopsis* lines (WT, *dgat1*, and *pdat1*) incorporated a similar amount of ^14^C into the total glycerolipid fraction (Supplementary Fig. S3 at *JXB* online). However, the rate of [^14^C]12:0 incorporation into TAG by leaves of *dgat1* was 4-fold lower than that of the WT during the 60min pulse (Fig. 1A). As noted in the Introduction, there are at least eight *Arabidopsis* genes that encode enzymes with DGAT activity. A corollary conclusion from the 76% reduction of TAG synthesis in *dgat1* is that other candidate DGAT activities cannot compensate for the major loss of leaf TAG synthesis in the *dgat1* mutant. In contrast to the 76% reduction in *dgat1*, there was only a 22% reduction of TAG accumulation in the *pdat1* mutant background (Fig. 1A)

**Fig. 2.**
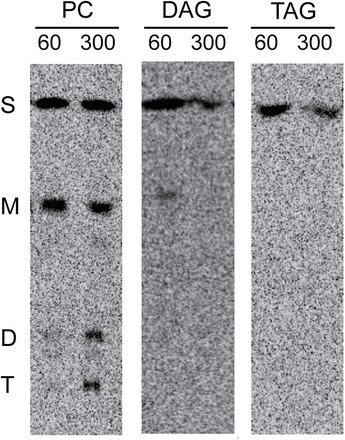
Unsaturated [^14^C]fatty acids are not incorporated into triacylglycerol after a pulse–chase labelling experiment. Fatty acid methyl esters after 60min (end of pulse) and 300min (end of chase) of PC, DAG, and TAG separated by argentation TLC. Each lane was loaded with radioactivity corresponding to 10 kdpm. PC, phosphatidylcholine; DAG, diacylglycerol; TAG, triacylglycerol; S, saturated FA; M, mono-unsaturated FA; D, di-unsaturated FA; T, tri-unsaturated FA. Representative autoradiograms of TLC plates are shown.

The combination of a 4-fold reduction of [^14^C]TAG synthesis in *dgat1* compared with the WT and the inability of PDAT1 or other possible acyltransferase enzymes to compensate for this loss supports the conclusion that DGAT1 is the major enzyme responsible for TAG synthesis in young *Arabidopsis* leaves. The possibility cannot be ruled out that other enzymes mentioned above could be responsible for the remaining level (24%) of TAG synthesis in *dgat1* or that these enzymes play a more substantial role under other conditions such as stress or senescence (e.g. Lippold *et al.*, 2012). It should also be noted that radiolabel must first enter PC before it can be a substrate for [^14^C]TAG synthesis by PDAT. Therefore, at early incubation time points, [^14^C]12:0 incorporation via DGAT activity may be favoured over PDAT. However, essentially no ^14^C-labelled unsaturated FAs in PC were transferred to TAG over the 5h time course (Fig. 2), indicating limited PDAT contribution to TAG synthesis in these leaves.

### Accumulation of ^14^C-labelled TAG is transient

Pulse–chase labelling provided the ability to monitor the turnover of ^14^C-labelled lipids. After removal of [^14^C]12:0 from the incubation medium, radiolabel in [^14^C]TAG decreased 70% during the 4h chase period, with a similar decrease in DAG (Fig. 1A, B). In contrast, radiolabel in PC declined by only 35% (Fig. 1C) (see Supplementary Fig. S2 at *JXB* online for TLC analysis). In contrast, there was little or no loss of label from the major chloroplast lipid monogalactosyldiacylglycerol (MGDG) (Supplementary Fig. S3), indicating that chloroplast lipids have a lower turnover rate than extraplastidial lipids (consistent with Pollard and Ohlrogge, 1999). The total radiolabel in all major and minor lipid classes was reduced ~20% during the chase period, indicating that DAG and TAG were the major lipid species that lost radiolabel.

Acyl transfer and acyl exchange are reversible reactions (Stymne and Stobart, 1984; Lager *et al.*, 2013) and, therefore, in addition to lipases, acyltransferases could provide an enzymatic mechanism for removal of acyl chains from TAG. Therefore, the reverse reactions of DGAT and PDAT represent candidates for enzymes that remove FA from the *sn*-3 position of TAG. However, the overall loss of radiolabel from [^14^C]TAG in *dgat1* and *pdat1* mutants was similar to that in the WT (Fig. 1), indicating that reversibility of DGAT or PDAT plays a limited role in the observed [^14^C]TAG turnover.

The pulse-labelling studies above indicate a rapid synthesis of TAG in leaves, and the chase period reveals a substantial turnover/degradation of the [^14^C]TAG. The fate of acyl chains released from TAG is unknown. Because the level of ^14^C-labelled PC or MGDG or other glycerolipids did not increase during the chase period, there was not a net transfer of [^14^C]acyl chains from TAG or DAG to other lipids. The observed degradation of [^14^C]TAG could be a consequence of β-oxidation of the labelled acyl chains. To test this hypothesis, pulse–chase labelling was also preformed on the *pxa1/cts/ped3 Arabidopsis* mutant, which is defective in a peroxisomal ABC transporter (PXA1) responsible for importing FAs (or acyl-CoA) into the peroxisome (Zolman *et al.*, 2001; Graham, 2008). There was no significant difference in synthesis or turnover of [^14^C]TAG between WT and *pxa-1* leaves (Supplementary Fig. S3 at *JXB* online). Studies on other β-oxidation mutants (*kat2*, *lacs6/7*, and *acx1/2*) during leaf senescence also showed no significant difference in FA turnover compared with WT plants (Yang and Ohlrogge, 2009). However, mutants of *COMATOSE* (*cts2*) accumulated more TAG after expression of *LEC2* in senescing leaves (Slocombe *et al.*, 2009). These different results may reflect redundancies or that 12:0 FFA can enter peroxisomes without participation of the PXA transporter (Linka and Theodoulou, 2013).

### Different metabolic fates for unmodified and plastid-modified [^14^C]acyl chains: [^14^C]12:0 is incorporated into TAG without elongation whereas plastid-modified [^14^C]FAs are incorporated into PC, DAG, MGDG, and PE, but not TAG

**Fig. 3.**
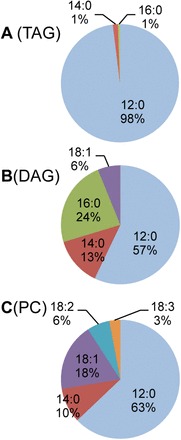
Distinct differences in [^14^C]fatty acid composition of triacylglycerol (TAG) compared with diacylglycerol (DAG) and phosphatidylcholine (PC). *Arabidopsis* wild-type (WT) leaves were labelled with [^14^C]12:0 for 60min. [^14^C]FA composition determined by argentation and reverse-phase TLC is shown for: (A) TAG, (B) DAG, and (C) PC. Data represent the average of three biological replicates. Tabulated averages ±SD are presented in Supplementary Table S1 at *JXB* online. (Figure 3 is available in colour at *JXB* online.)

As noted, exogenously supplied [^14^C]12:0 can be incorporated directly into cytosolic glycerolipids in addition to entering the plastid, where it is elongated and desaturated prior to incorporation into glycerolipids. This provided the ability to monitor simultaneously leaf TAG synthesis from cytosolic unmodified FA ([^14^C]12:0) and from FA exported from the plastid. To distinguish between these two pathways, the [^14^C]acyl chains incorporated into TAG, DAG, PC, phosphatidylethanolamine (PE), and MGDG were identified by reverse-phase TLC and/or argentation TLC (Fig. 3; Supplementary Fig. S4 at *JXB* online). Approximately 40% of the [^14^C]12:0 that entered all glycerolipid classes was modified by elongation and/or desaturation. However, the metabolic fates of the unmodified and the plastid-modified [^14^C]12:0 were strikingly different. Almost all (>99%) radioactivity of the plastid lipid MGDG was C16 or C18 FAs, with undetectable levels of [^14^C]12:0. In striking contrast, analysis of the [^14^C]acyl chains in TAG indicated that these were almost exclusively (98%) [^14^C]12:0 (Fig. 3A). The eukaryotic membrane lipids PC and PE contained [^14^C]12:0 and also substantial amounts of elongated saturated and unsaturated [^14^C]FA (Fig. 3C; Supplementary Fig. S4). Specifically, [^14^C]16 and [^14^C]18 products of chloroplast reactions represented 30% and 27% of the radiolabel in DAG and PC, respectively (Fig. 3B, C).

The markedly different composition of [^14^C]acyl groups in PC and DAG compared with TAG indicated that the [^14^C]FA that enters the plastid is modified, and then exported for PC and DAG synthesis and is not available for TAG synthesis. Furthermore, during the 4h chase, no elongated [^14^C]FAs were chased into TAG, indicating that the lack of modified [^14^C]FAs in TAG is not due to a lag through subcellular pools (Fig. 2).

### Evidence for multiple pools of extraplastidial acyl-CoA

After elongation in the plastid, FAs are exported and activated to acyl-CoA at the chloroplast envelope (Ohlrogge and Browse, 1995), after which they are incorporated into eukaryotic lipids. As noted above, the [^14^C]acyl chain composition of TAG was almost exclusively [^14^C]12:0, whereas [^14^C]PC and [^14^C]DAG were much more highly enriched in elongated/desaturated [^14^C]acyl chains compared with [^14^C]TAG (Fig. 3). The distinctly different labelling patters can be most readily explained by the presence of more than one acyl-CoA pool responsible for their synthesis. An alternative hypothesis of a single [^14^C]acyl-CoA pool would require differences in specificity of acyltransferases or other enzymes that synthesize these glycerolipids (or their precursors) to explain the different composition of [^14^C]FA in PC and TAG. DGAT1 is responsible for >75% of leaf [^14^C]TAG synthesis (Fig. 1A). Therefore, in the case of TAG, the accumulation of [^14^C]12:0 at 98% and almost complete absence of [^14^C]16:0, [^14^C]18:1, and [^14^C]14:0 would require DGAT1 to be strongly selective for [^14^C]12:0 in an acyl-CoA pool that contains a mixture of [^14^C]12:0 and the elongated/desaturated [^14^C]acyl chains. Previous analyses of DGAT activity with different [^14^C]acyl chains have not indicated strong selectivity for [^14^C]12:0 over [^14^C]16:0, [^14^C]18:1, and [^14^C]14:0 that could account for these results (Lung and Weselake, 2006; Snyder *et al.*, 2009). Likewise, other acyltransferases involved in glycerolipid biosynthesis such as PDAT, LPCAT, and LPEAT show relatively little acyl-CoA selectivity (Ståhl *et al.*, 2004, 2008; Stålberg *et al.*, 2009). As a result, the distinct labelling patterns of TAG and PC are best explained by the occurrence of different substrate pools of acyl-CoA that provide acyl chains for their synthesis. This conclusion is also consistent with recent kinetic studies of export of FAs from chloroplasts and their incorporation into PC (Bates *et al.*, 2007; Tjellström *et al.*, 2012) where rapid incorporation of newly synthesized [^14^C]acyl chains into PC was inconsistent with their mixing with a bulk pool of cellular acyl-CoA. The occurrence of distinct cellular acyl-CoA pools has also been proposed as an explanation for differential specificity of fatty alcohol reductase (FAR) enzymes when expressed in different organisms (Doan *et al.*, 2009).

### All eukaryotic DAGs are not available as a substrate for DGAT1

The occurrence of spatially distinct pools of DAG in plant cells has been proposed based on enzyme assays (Vogel and Browse, 1996), and labelling studies and subcellular fractionation (Siebertz *et al.*, 1979; Vogel and Browse, 1996; Bates *et al*., 2009, 2012). The FA composition of DAG in general resembles the *sn*-1 and *sn*-2 positions of TAG. This pattern has been observed in many seeds (Bates *et al.*, 2009; Bates and Browse, 2011), including transgenic high laurate *Brassica napus* (Wiberg *et al.*, 2000). However, data presented in Fig. 3 indicate that the radiolabelled DAG and TAG pools from leaves have a distinctly different [^14^C]acyl chain composition. In particular, the level of 16- and 18-carbon [^14^C]acyl chains in DAG is >10-fold higher than in TAG (Fig. 3). These data imply that there is not a direct precursor–product relationship between the [^14^C]DAG pool that contains [^14^C]16:0 and [^14^C]18:1, and the pool that supplies [^14^C]TAG products. Using the same reasoning discussed above for multiple acyl-CoA pools, these results could be explained by either (i) enzyme(s) that are selective for [^14^C]12:0-enriched DAG and/or against [^14^C]16:0 and [^14^C]18:1 DAG; or (ii) multiple pools of DAG. Data on DGAT assays on substrate selectivity in safflower and *B. napus* microsomes indicate that DGAT does not have substrate selectivity for the composition of DAG that could account for the exclusion of [^14^C]16:0 and [^14^C]18:1 acyl chains in TAG (Ichihara and Noda, 1982; Vogel and Browse, 1996; Lung and Weselake, 2006). Therefore, the distinct differences in [^14^C]DAG and [^14^C]TAG composition observed here provide *in vivo* evidence for distinct DAG pools as the most probable explanation for the major difference in [^14^C]acyl composition of DAG and TAG.

**Fig. 4.**
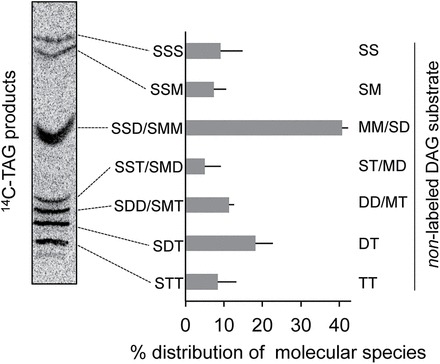
**[** ^12^C]Diacylglycerel (DAG) molecular species precursor for [^14^C]triacylglycerol (TAG) synthesis. Molecular species of [^14^C]TAG were separated by argentation TLC. The major [^14^C]TAG species contain two double bonds (MMS or SSD). Because all radiolabelled [^14^C]FAs in TAG are saturated (Fig. 3A), the molecular species of the unlabelled [^12^C]DAG precursor of [^14^C]TAG must also possess two double bonds (MM or SD). The data represent the average of three biological replicates. S, saturated FA; M, mono-unsaturated FA; D, di-unsaturated FA; T, tri-unsaturated FA. No regiochemistry is specified. A representative TLC plate is shown.

Further support for distinct DAG pools was obtained by argentation TLC analysis of the molecular species of [^14^C]TAG. The major [^14^C]TAG species contain two double bonds {MMS or SSD [saturated FA (S), mono-unsaturated FA (M), and di-unsaturated FA (D)]} representing 40% of all species (Fig. 4). Because all radiolabelled [^14^C]FAs in TAG are saturated (Fig. 3A), the molecular species of the unlabelled [^12^C]DAG precursor of [^14^C]TAG must also possess two double bonds (MM or SD). This [^12^C]DAG composition is not consistent with the expected eukaryotic DAG molecular species in leaves. First, ~77% of DAG molecular species in *Arabidopsis* leaves do not have two double bonds. The predominant species with two double bonds is SD (16:0/18:2) and represents only ~23% (Vom Dorp *et al*., 2013). Although these data do not distinguish the distribution of DAG between plastid and extraplastidial membranes, the low level of 16:0/18:1 molecular species and almost complete absence of 16:3 species implies that most leaf DAG is extraplastidial. Secondly, PC and DAG are considered to be in equilibrium in leaves (via the activity of CDP-choline transferase and/or PDCT), and therefore DAG molecular species reflect the molecular species of eukaryotic DAG (Slack *et al.*, 1985). Data on the PC molecular species of *Arabidopsis* leaves indicate that most PC species have more than two double bonds and only 14% of all PC species have two double bonds (M. Li *et al.*, 2006). The difference between [^12^C]DAG precursor of [^14^C]TAG (Fig. 4) and the eukaryotic DAG pool (inferred from PC and the total DAG pool in leaves) provides a second line of evidence that, as in seeds (Bates *et al.*, 2009), a metabolically distinct subpool of eukaryotic DAG is present in leaves.

Although distinct metabolite pools can explain the data reported here, the hypothesis (as that from other studies) is inferred from indirect evidence, namely radiolabelling. Acquiring direct evidence of the subcellular distribution of metabolites involved in lipid synthesis and assembly has been difficult. Subcellular fractionation generally requires times longer than the half-life of acyl-CoA and other metabolites, and can result in mixing and cross-contamination of cytosol and organelles. Clearly, a more precise understanding of the subcellular localization of substrates in lipid metabolism and to what extent these substrates are involved in metabolic channelling is a key but challenging task.

It should be noted that DAG or acyl-CoA subpools may require little spatial separation if there is metabolic channelling or subdomains of the endoplasmic reticulum (ER) or other membranes (Andersson *et al.*, 2007; Cahoon *et al.*, 2007; Benning, 2009; Mehrshahi *et al.*, 2013). In addition, DGAT1 is a membrane-spanning protein localized in the ER. Experiments on animal DGAT indicate that at least 50% of the activity is associated with the lumen leaflet of the ER, and that amino acid residues required for DGAT activity face the ER lumen (McFie *et al.*, 2010; Wurie *et al.*, 2011). If this were also the case for *Arabidopsis*, DAGT1 may use an acyl-CoA and/or DAG pool located in the lumen or the ER membrane facing the lumen rather than in the cytosol or other membranes.

It was also taken into consideration that some of the observed differential labelling of PC, DAG, and TAG might reflect different cell types, for example epidermal and mesophyll. However, the observed labelling patterns are more consistent with labelling of mesophyll cells rather than epidermal cells. MGDG was a major labelled lipid and is an abundant mesophyll but not epidermal lipid. Furthermore, no wax products characteristic of epidermal lipid synthesis were observed from the labelling.

It also cannot be ruled out that synthesis of TAG at other stages of development or in leaves that have been engineered to produce high levels of TAG will utilize different metabolic pathway with different substrates and/or enzymes. The origin of DAG and acyl-CoA pools used for leaf TAG synthesis in these cases should also be further investigated by labelling *de novo* FA synthesis with [^14^C]acetate or ^14^CO_2_.

In conclusion, pulse–chase labelling using [^14^C]12:0 enabled measurement of TAG synthesis and turnover in leaves. Using this methodology, it is shown that, as in *Arabidopsis* seeds (Zhang *et al.*, 2009), DGAT1 is the primary enzyme involved in TAG synthesis in young leaves. In this study it was possible to trace leaf glycerolipid synthesis concurrently both from cytosolic unmodified ([^14^C]12:0) FA and from acyl chains exported from the plastid after elongation and desaturation by plastid enzymes. [^14^C]12:0 was rapidly incorporated into PC, DAG, and TAG. In contrast, [^14^C]16:0 and [^14^C]18:1 derived by plastid elongation and desaturation were incorporated into plastid MGDG, PC, and DAG, but not TAG. The distinct differences in the [^14^C]acyl chain composition of PC, DAG, and TAG provide *in vivo* evidence that there are subpools of acyl-CoA involved in the synthesis of these different extraplastidial glycerolipids. In addition, it is proposed that leaf TAG is synthesized using a specific DAG subpool because [^14^C]acyl chains that are elongated/desaturated in the plastid were incorporated into PC and DAG, but not TAG.

These results re-emphasize that plant lipid metabolism involves a level of subcellular compartmentation that extends to separate metabolic pools of central intermediates, including DAG and acyl-CoA, that participate in multiple pathways. This information may help interpret results from and guide strategies for metabolic engineering of lipid metabolism. For example, the common observation that unusual FAs of seeds are excluded from membrane lipids in native species, but not in transgenics (Millar *et al.*, 2000; Cahoon *et al.*, 2007), may suggest that a heterologously introduced acyl modification enzyme must be targeted so that it acts exclusively on intermediates that are compartmentalized or channelled for TAG biosynthesis.

## Supplementary data

Supplementary data are available at *JXB* online.

Figure S1. Relative incorporation of [^14^C]12:0, 16:0, or 18:1 fatty acids into WT *Arabidopsis* leaves.

Figure S2. Representative TLC plates of [^14^C]12:0-labelled lipids isolated from WT *Arabidopsis*.

Figure S3. Incorporation of ^14^C into major lipids classes in Col-0, *pdat1*, *dgat1*, and *pxa1.*

Figure S4. Reverse-phase TLC of ^14^C-labelled acyl chains in glycerolipids after 60min of [^14^C]lauric acid labelling.

Figure S5. ^14^C-Labelled molecular species of TAG, DAG, and PC.

Table S1. [^14^C]FA composition of TAG, DAG, and PC.

Supplementary Data

## Abbreviations:

DAG: diacylglycerol
DGAT: diacylglycerol acyl transferase
FA: fatty acid
FFA: free fatty acid
PC: , phosphatidylcholine
TAG: triacylglycerol.
