# Supplementary material for: Tracking synthesis and turnover of triacylglycerol in leaves
Source: J Exp Bot. 2015 Jan 21;66(5):1453–61. doi: 10.1093/jxb/eru500 (PMC4339603; doi:10.1093/jxb/eru500)
Supplement: Supplementary Data [file supp_66_5_1453__index.html]

Tracking synthesis and turnover of triacylglycerol in leaves — Tracking synthesis and turnover of triacylglycerol in leaves — Supplementary Data 

# Tracking synthesis and turnover of triacylglycerol in leaves

## Supplementary Data

Data files

**Files in this Data Supplement:**

- Supplementary Data - Supplementary Data
