# Supplementary material for: Tracking synthesis and turnover of triacylglycerol in leaves
Source: J Exp Bot. 2015 Jan 21;66(5):1453–61. doi: 10.1093/jxb/eru500 (PMC4339603; doi:10.1093/jxb/eru500)
Supplement: Supplementary Data [file supp_eru500_jexbot138016_file001.pdf]

Supplemental figure 1

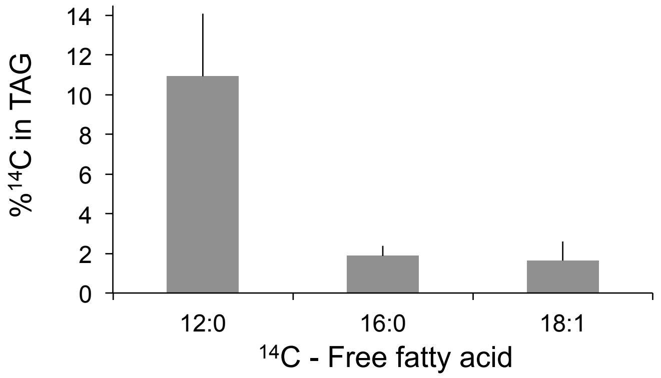

Supplemental figure 1. Relative incorporation of ^{14}C-fatty acids into triacylglycerols in WT. Equal amounts of ^{14}C labeled 12:0, 16:0 or 18:1 were added to leaves and incubated for 60 min prior to lipid extraction and TLC**.** Data points represent average ± SD from 3 biological replicates.

Supplemental figure 2

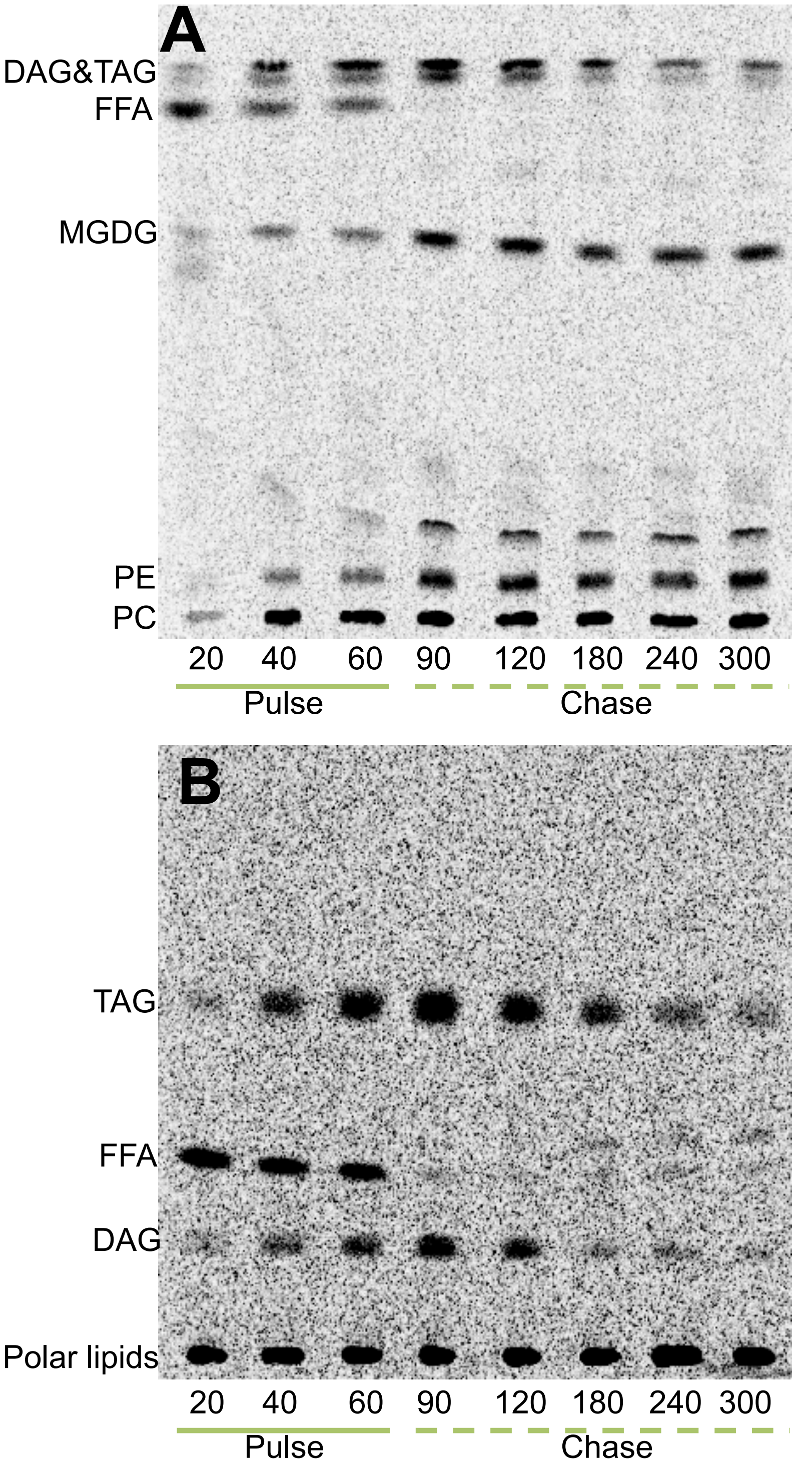

Supplemental figure 2: Representative autoradiograms of TLC plates of ^{14}C-12:0 labeled lipids isolated from WT leaves

Lipids equivalent to 3µg chlorophyll loaded in each lane. A, polar TLC developed with acetone:toluene:water (91:30:8). B, Neutral TLC developed hexane:diethylether:acetic acid (70:30:1) DAG: Diacylglycerol; FFA: Free fatty acid PC:Phosphatidylcholine; PE:Phosphoethanolamine (PE); MGDG: Monogalactosyldiacylglycerol and Triacylglycerol (TAG).

Supplemental figure 3

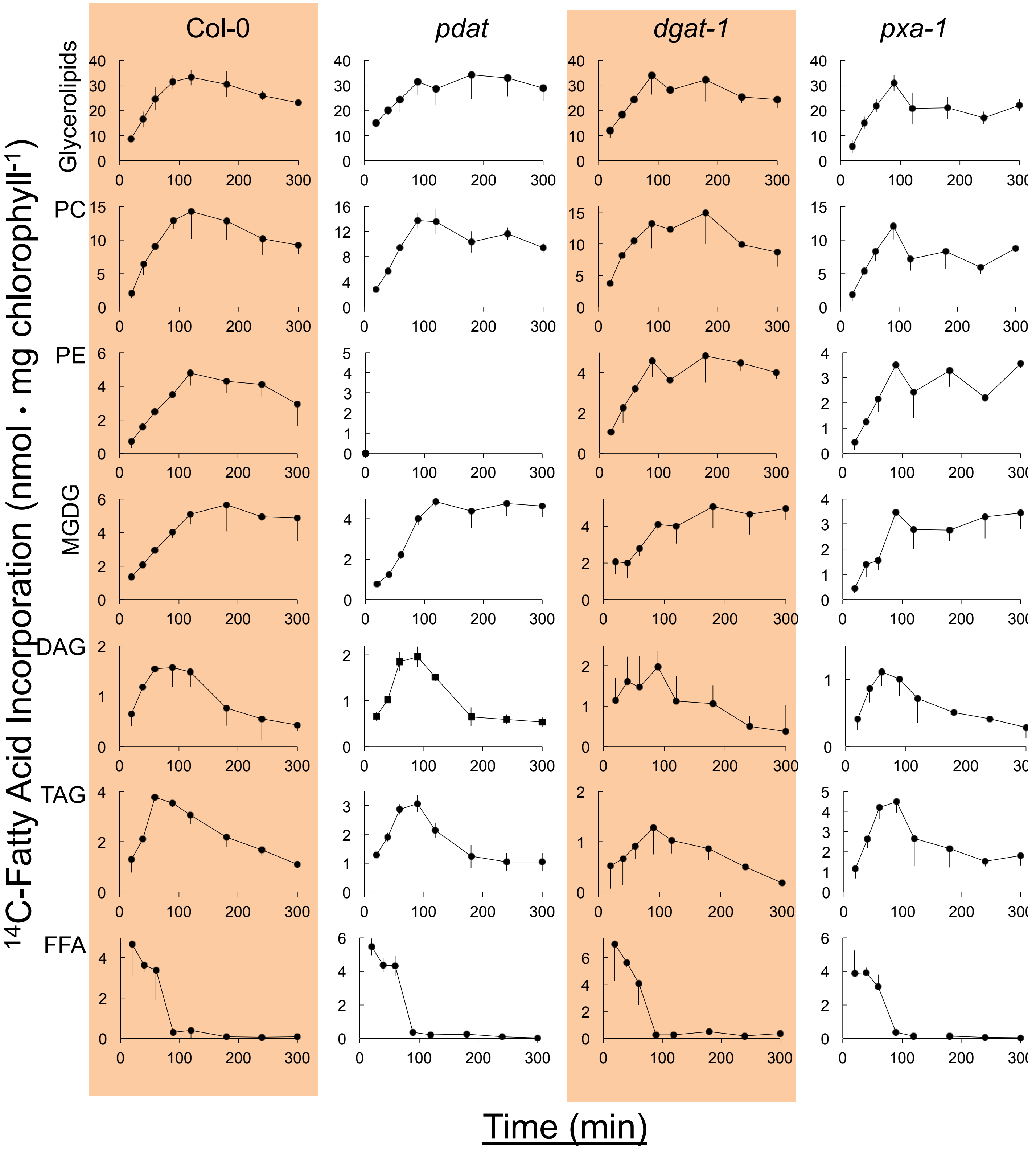

Supplemental figure 3: Side by side comparison of ^{14}C abundance in lipids in WT, *pdat*, *dgat1* and *pxa-1*
DAG: Diacylglycerol; FFA: Free fatty acid PC: Phosphatidylcholine; PE: Phosphatidylethanolamine; MGDG: Monogalactosyldiacylglycerol and Triacylglycerol (TAG). Data represent average ± SD from 3 biological replicates

Supplemental figure 4

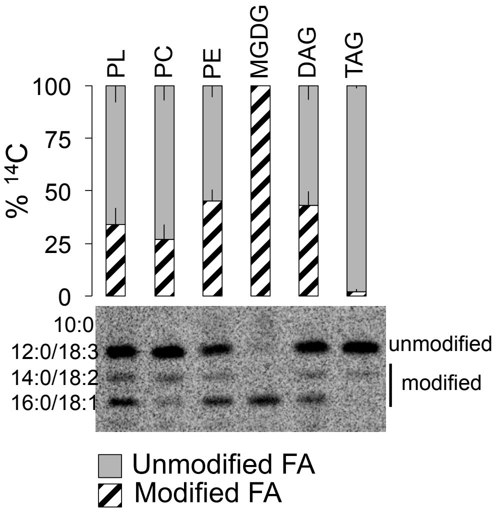

Supplemental figure 4. Membrane lipids but not TAG contain plastid modified ^{14}C-fatty acid
Histogram presents the proportions of radioactivity in fatty acids (analyzed as FAMEs) after 60 min of ^{14}C-lauric acid pulse labeling. Autoradiogram presents a representative reverse phase TLC plate (10000 DPM loaded in each lane). Polar lipids (PL); Phosphatidylcholine (PC); Phosphoethanolamine (PE); Monogalactosyldiacylglycerol (MGDG); Diacylglycerol (DAG); Triacylglycerol (TAG).

Supplemental figure 5

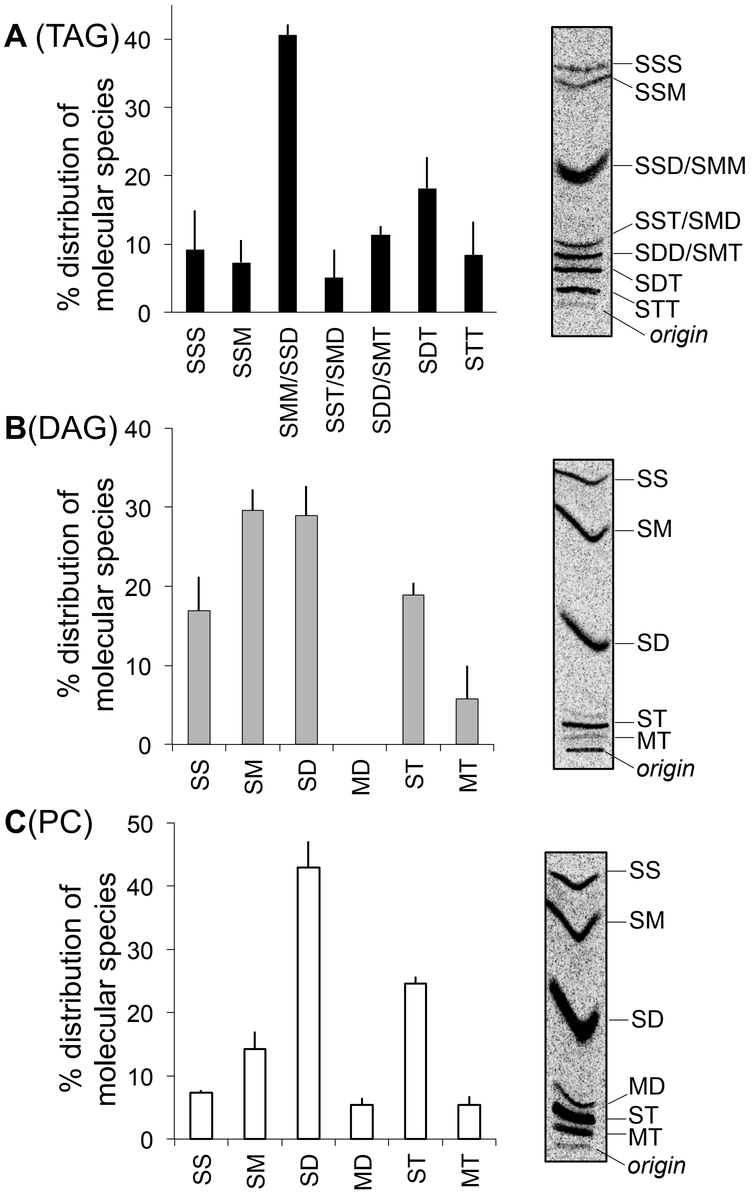

Supplemental figure 5. Molecular species of ^{14}C-labled TAG, DAG and PC after 60 min of ^{14}C-12:0 labeling of WT leaves

Saturated FA (S); Mono-unsaturated FA (M); Di-unsaturated FA (D) and Tri-unsaturated FA (T). Data represent average ± SD of 3 biological replicates. No regiochemistry is specified

Supplemental table I: ^{14}C-fatty acid composition of triacylglycerol (TAG), diacylglycerol (DAG) and phosphatidylcholine (PC). Data represent % 14C distribution within each lipid ± SD of three biological replicates.

Fatty acid	TAG	DAG	PC
12:0	98.1±1.3	57.1±6.9	63.5±1.3
14:0	1.4±1.2	13.2±3.3	9.6±2.1
16:0	0.5±0.5	23.5±4.5	-0.9±1.5
18:1	*nd*	6.2±0.2	18.6±1.2
18:2	*nd*	*nd*	6.2±2.1
18:3	*nd*	*nd*	3.0±0.7

nd= not detected
